# 

**Published:** 1875-10

**Authors:** 


### Contents of October Number.

### ORIGINAL.

ART. I.—On Unreduced Dislocations of the Shoulder. By Edward N. Brush, M. D., ... 81
ART. II.—Chorea. By Henry Nickell, M. D., ... 100

### MISCELLANEOUS.

Unsuccessful Practitioners, ... 102
Treatment of Fractures by means of Plaster of Paris, ... 104
Enteric Fever and Milk Supply, ... 107
Treatment of Chronic Cystitis ... 109
Chloralism, ... 111
Mr. Teale's Case of High Temperature ... 113
Duration of Bloodless Operations, ... 113
Poisoning by Camphor, ... 114
Correspondence, ... 115

### EDITORIAL DEPARTMENT.

Unsuccessful Practitioners, ... 115
Life Insurance Companies and Physicians, ... 119

BOOKS REVIEWED:

Zimssen's Cyclopædia, Vol. X, ... 117
Vision: Its Optical Defects. By C. S. Fenner, M. D., ... 119

Books and Pamphlets Received, ... 120

